# Treatment Options and Outcomes of Penile Constriction Devices

**DOI:** 10.1590/S1677-5538.IBJU.2018.0667

**Published:** 2019-04-01

**Authors:** Leandro Koifman, Daniel Hampl, Maria Isabel Silva, Paulo Gabriel Antunes Pessoa, Antonio Augusto Ornellas, Rodrigo Barros

**Affiliations:** 1 Hospital Municipal Souza Aguiar, Rio de Janeiro, RJ, Brasil;; 2 Instituto Nacional do Câncer (INCA), Rio de Janeiro, Brasil

**Keywords:** Penis, Constriction, Therapeutics

## Abstract

**Purpose:**

To study the effect of penile constriction devices used on a large series of patients who presented at our emergency facility. We explored treatment options to prevent a wide range of vascular and mechanical injuries occurring due to penile entrapment.

**Materials and Methods:**

Between January 2001 and March 2016, 26 patients with penile entrapment were admitted to our facility and prospectively evaluated.

**Results:**

The time that elapsed from penile constrictor application to hospital admission varied from 10 hours to 6 weeks (mean: 22.8 hours). Non-metallic devices were used by 18 patients (66.6%) while the other nine (33.4%) had used metallic objects. Acute urinary retention was present in six (23%) patients, of whom four (66.6%) underwent percutaneous surgical cystotomy and two (33.4%) underwent simple bladder catheterization. The main reason for penile constrictor placement was erectile dysfunction, accounting for 15 (55.5%) cases. Autoerotic intention, psychiatric disorders, and sexual violence were responsible in five (18.5%), five (18.5%), and two (7.4%) cases, respectively. The mean hospital stay was 18 hours (range, 6 hours to 3 weeks).

**Conclusion:**

Penile strangulation treatment must be immediate through the extraction of the foreign body, avoiding vascular impairments that can lead to serious complications. Most patients present with low-grade injuries and use penile constrictors due to erectile dysfunction. Removal of constrictor device can be challenging. The use of specific tools for achieving penile release from constrictors is a fast, safe and effective method. Patients with urinary retention may require urinary diversion.

## INTRODUCTION

Penile strangulation is a rare emergency situation that requires proper immediate intervention to prevent a wide range of vascular and mechanical injuries. First reported in 1755 by Gaultier (1), there are many reports of penile incarceration in the international literature and most of them are single case reports.

In middle-aged and elderly men, the leading cause of penile injury by foreign bodies is the attempt to increase sexual performance or due to autoerotic intentions (2), while masturbation and sexual curiosity are the leading causes in male adolescents. In infants and children, the foreign body is usually a string (3), thread (4) or hair tied around the penis. Psychiatric disorders can occur at any group age, leading patients to the application of penile constrictors and therefore incarceration.

A variety of objects of different nature (metallic and nonmetallic) have been used for this purpose, all with a similar feature, circularity. Entrapment of the penis by an encircling object leads to a range of vascular injuries that begins with penile swelling distal to the object due to the initial blockage of venous and lymphatic return. After a few hours of incarceration, compartmental syndrome arises that may result in tissue necrosis and gangrene, especially when associated with arterial obstruction. A variety of mechanical injuries can be inflicted upon the entrapped penis, including skin ulceration, urethral injuries, constriction of the corpus spongiosum and corpora cavernosa, urethral fistula development, and loss of distal penile sensation (5–7).

Penile entrapment is usually challenging to urologists working in the emergency room and surgeons must creatively use the best medical instruments and ordinary tools available in hospital facilities. Sometimes non-medical professionals are called and can be very helpful.

The aim of this study is to evaluate the clinical findings, treatment options, complications and outcomes of a large series of patients who presented at our emergency facility with penile entrapment.

## MATERIAL AND METHODS

Between January 2001 and March 2016, 26 patients (27 cases; one patient had two episodes in a 45-day interval) with penile entrapment were admitted to our facility and prospectively evaluated.

The primary diagnostic assessment consisted of clinical history and physical examination. According to our hospital infection committee, first-generation cephalosporin was administered in all cases and a tetanus prophylaxis was given when needed.

We analyzed the time that elapsed from penile constrictor application to the hospital admission, presence of urinary retention, type of constrictor used, treatment options, early and late outcomes, and hospitalization period.

We classified the penile constrictor devices into type (metallic or non-metallic), motivation behind its use (erectile dysfunction, autoerotic, psychiatric disorders, and sexual violence), and complications according to the Bhat grading system and Silberstein modified categories (8, 9) (Table-1).

**Table 1:** Treatment options for penile entrapment according to penile constrictor device and constriction injuries classification according to Bhat grading system and Sylberstein modified categories.

Penile constrictor classification (N)	Number of cases (%)	Type of constrictor	Bhat's grade system (N)	Silberstein modified categories (N)	Treatment tool option	
					Instrument options	Nunber cases
Nonmetallic (18)	07 (26%)	Pet Bottle	Grade I (4)	Low-grade (4)	Lister scissors	01
					Gigli saw	02
			Grade II (2)	Low-grade (2)	Dental drill	03
					Orthopedics cutting pliers	04
			Grade III (2)	Low-grade (1)		
	04 (14.8%)	Plastic tube	Grade I (2)	Low-grade (2)	Lister scissors	01
			Grade II (1)	Low-grade (1)	Gigli saw	01
					Dental drill	01
			Grade III (1)	Low-grade (1)	Orthopedics cutting pliers	01
	03 (11.1%)	PVC tube	Grade I (2)	Low-grade (2)	Gigli saw	03
			Grade II (1)	Low-grade (1)		
	02 (7.4%)	Hair	Grade I (1)	Low-grade (1)	Lister scissors	02
			Grade V (1)	High-grade (1)		
	02 (7.4%)	Plastic ring	Grade V (2)	High-grade (2)	Gigli saw	02
Metallic (9)	04 (14.8%)	Metallic ring	Grade I (3)	Low-grade (3)	Dental drill	01
					Orthopedics cutting pliers	02
				Low-grade (1)	Eletric saw	01
	03 (11.1%)	Aluminum tube	Grade I (2)	Low-grade (2)	Orthopedics cutting pliers	03
		Grade II (1)	Low-grade (1)			
			Grade I (1)	Low-grade (1)	Eletric saw	02
	02 (7.4%)	Gear nut	Grade II (1)	Low-grade (1)		

Early and late complications were defined as those that emerged within and after 30 days of penile constrictor removal, respectively.

Patients were followed at outpatient weekly visits in the first month after hospital discharge and then 4 / 4 months during the first year.

Our institutional review board approved the study. The mean follow-up was 11.8 months.

## RESULTS

**Figure 1:**
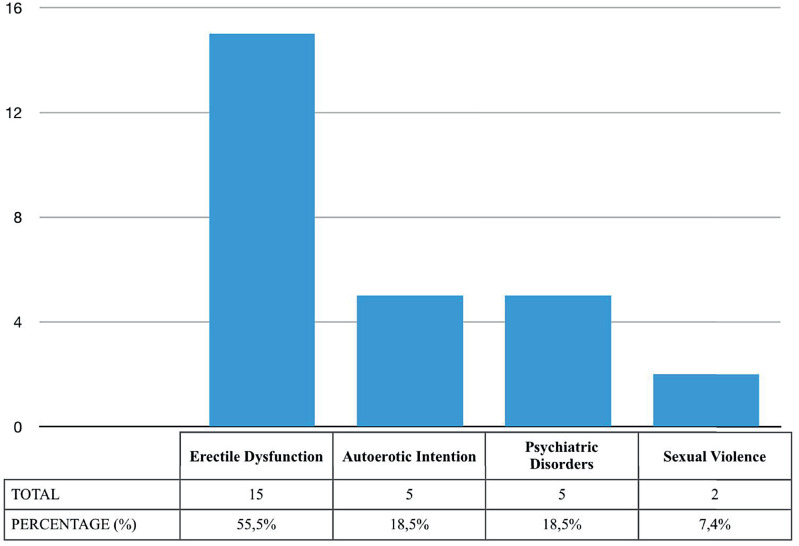
Penile constrictor application according to its etiology.

The patient's ages ranged from 17-68 years (mean: 45.7 years). The time that elapsed from penile constrictor application to hospital admission varied from 10 hours to 6 weeks (mean: 22.8 hours). Non-metallic devices were used by 18 patients (66.6%) while the other nine (33.4%) had used metallic objects. Acute urinary retention was present in six (23%) patients, of whom four (66.6%) underwent percutaneous surgical cystostomy and two (33.4%) underwent simple bladder catheterization. The main reason for penile constrictor placement was erectile dysfunction, accounting for 15 (55.5%) cases. Autoerotic intention, psychiatric disorders, and sexual violence were responsible in five (18.5%), five (18.5%), and two (7.4%) cases, respectively (Figure-1). The mean hospital stay was 18 hours (range, 6 hours to 3 weeks).

The most frequent constrictor type used by the patients in this study was a polyethylene terephthalate (PET) bottle, accounting for seven (26%) cases, while the less frequent objects were plastic rings, hair, and gear nuts, each of which was responsible for two (7.4%) cases. Penile entrapment release was obtained using a variety of medical instruments and tools according to constrictor type (Table-1). In three (11.1%) cases, help from the fire brigade was needed to remove the penile constrictor. Penile entrapment release was performed under spinal anesthesia in 03 cases (11.2%), local anesthesia in 12 cases (44.4%) and in the remaining 12 cases (44.4%) there was no need for any type of anesthesia.

Of the 27 cases evaluated, according to Bhat grading system, 15 (55.5%) consisted of grade I injuries, seven (26%) consisted of grade II injuries, and two (7.4%) consisted of grade III injuries. The remaining three (11.1%) cases involved grade V penile constrictor injuries. When stratifying patients according to Bhat's grading system for Silberstein classification, it was possible to identify 24 (88.8%) cases of low-grade lesions and three (11.2%) of high-grade lesions (Table-1).

The early complications included edema in 25 (92.5%) cases, necrosis and skin infection in two (7.4%) cases, and decrease / loss of penile sensation, abscess / cellulite, penile amputation, and urethra fistula in one (3.7%) case each. The late complications included decreased / lost penile sensation in one (3.7%) patient and urethral stricture in two (7.4%) cases (Table-2).

**Table 2:** Early and late complications after penile constrictor release.

Complication	No. Complication (%)	
	Early	Late
Edema	25 (92.5)	0
Wound infection	2 (7.4)	0
Loss penile sensation	1 (3.7)	1 (3.7)
Cellulite	1 (3.7)	0
Penile amputation	1 (3.7)	0
Urethral fistula	1 (3.7)	0
Urethral stricture	0	2 (7.4)

After hospital discharge, all patients were referred to our sexual dysfunction department for follow-up and evaluation.

## DISCUSSION

Penile strangulation is a rare urological emergency. The presentation of each case varies, and removing the constriction devices can present great challenges. A variety of types of metallic and non-metallic constriction devices have been used, ranging from simple plastic rings to rubber bands, hair, hammerheads, soft drink bottles, and, most frequently, various metal rings (9–11). In our series, the majority of patients (66.6%) used non-metallic constriction, while the others (33.4%) used metallic devices.

The complications of penile strangulation vary and depend on factors including type of device used, degree of constriction, and time elapsed until presentation. Several authors have attempted to grade such injuries. Bhat et al. developed a grading system for penile injuries and divided them into five categories ranging from penile edema to gangrene (8). Grade I causes only edema, while Grade II involves penile paresthesia. Grade III involves injury to the skin and urethra but no urethral fistula. Grade IV involves urethral fistula. Grade V injury involves gangrene, necrosis, or complete amputation. Further, Silberstein et al. simplified this grading system by modifying it into two broad categories (9). Low-grade injuries include penile edema, ulceration of skin, and decreased penile sensation with no evidence of a urethral fistula. High-grade injuries are defined as injuries that are likely to require surgical intervention. In our experience, the most common type of injury, according to the Bhat grading system, was grade I in 15 (55.5%) cases and only three (11.1%) cases of grade V. When stratifying patients according to Bhat's grading system for Silberstein classification, we found 24 (88.8%) cases of low-grade lesions and three (11.2%) cases of high-grade lesions (Table-1).

Regardless of the treatment option, the main objective is removal of the constricting device to restore venous and lymphatic drainage and arterial inflow, preserving the organ's anatomy and functionality. This approach must be delivered urgently since prolonged placement of constriction devices is considerably more likely to result in high-grade injuries. According to Broderick et al. in a study using color Doppler ultrasonography, penile incarceration > 30 minutes may result in penile ischemia (12). Despite this, the mean time that elapsed from penile constrictor application to hospital admission in our series was 22.8 hours.

**Figure 2:**
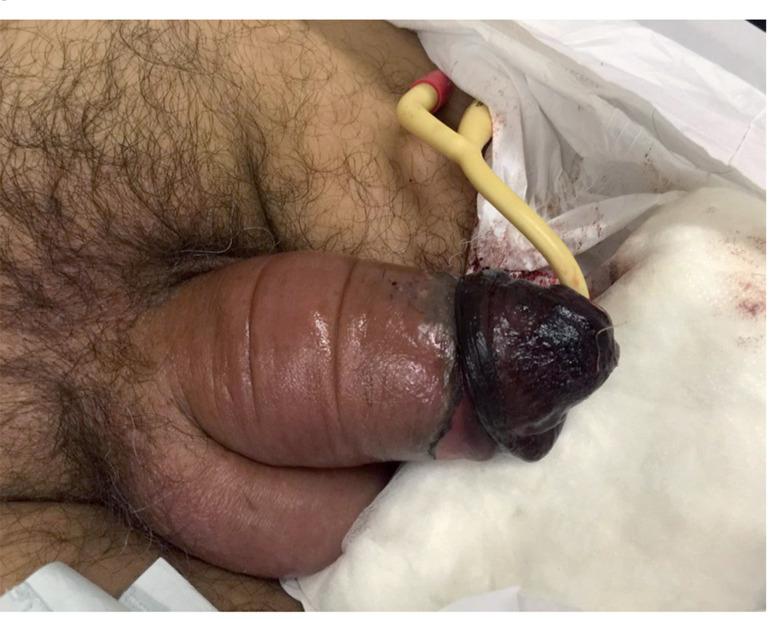
Patient with penile incarceration produced by a plastic ring with signs of tissue impairment of the distal penile shaft.

The main reasons for this delay are patient shame and psychiatric disorders. We had two cases in which penile amputation was required, both of which involved late presentation. In the first case, the patient was 16 years old and had a psychiatric disorder. He first used hair as a penile constrictor; after 45 days, he returned to our emergency department with a new penile strangulation for 48 hours when the distal end of the penis fell off due to necrosis. The patient was taken to the operating room for penile debridement. In the other case, the patient presented to our hospital after 72 hours of using a plastic ring as a constrictor. Even after device removal, the patient developed necrosis and infection of distal third of the penile shaft (Figure-2). After conservative treatment with antibiotics and debridement, the evolution was unfavorable and the patient underwent a partial penectomy.

The type of constricting device appeared to impact the degree of penile injury, with the more severe injuries induced by non-metallic devices. Non-metallic constricting devices accounted for 2 / 2 (100%) of high-grade penile injuries in our series, while metallic constrictors did not produce any high-grade lesions (0%). The increased elastic properties of non-metallic items makes them easier to position, but during the edema phase might be more likely to exert pressure on the penis, resulting in lesions of greater degree.

There are many reports of different devices that have been used as well as techniques and suggestions for their removal (13, 14). The approach of choice depends on the type of the constricting device, degree of injury, and available equipment (9). Penile aspiration could serve as the simple first step to reduce edema and provide more space to release the device (15). Katz et al. described a new noninvasive technique, the “pseudo-pulley” method, which involves the passage of four straight Nitinol hydrophilic guide wires to remove a penile constriction device (16). If non-invasive removal is not possible, an object may be cut or sawed off. Nonelectric cutting tools should be reserved for smaller and softer objects such as hair, plastic bottle rings, and smaller metal rings (17). Unfortunately, there are some reports of iatrogenic injury caused by these devices (18). Horstmann et al. reported the successful removal of a 3.6-cm long piece of heavy metal tubing using an angle grinder (19). In cases when all other extraction techniques have failed and there exists devitalized or gangrenous tissue, penile degloving and amputation can be employed (9).

**Figure 3:**
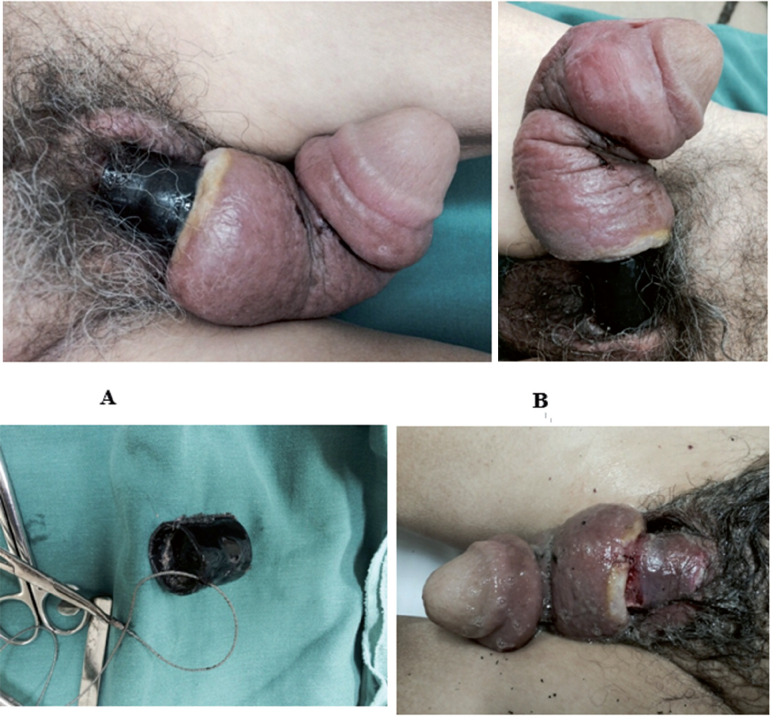
Patient with penile incarceration produced by plastic tube (A+B). Gigli saw used to remove the foreign body (C). Final appearance of the penile shaft after penile constrictor removal (D).

In our series, a Gigli saw (44.4%) was most commonly used to remove non-metallic penile constrictors, followed by orthopedic cutting pliers (22.2%). Gigli saws are manufactured with several interlaced micro-twisted and then braided steel strands that give them great cutting power. They are easily applied under the penile constrictor, allowing them to be removed quickly and non-invasively, constituting an excellent option for the removal of non-metallic constrictors devices (Figure-3).

**Figure 4:**
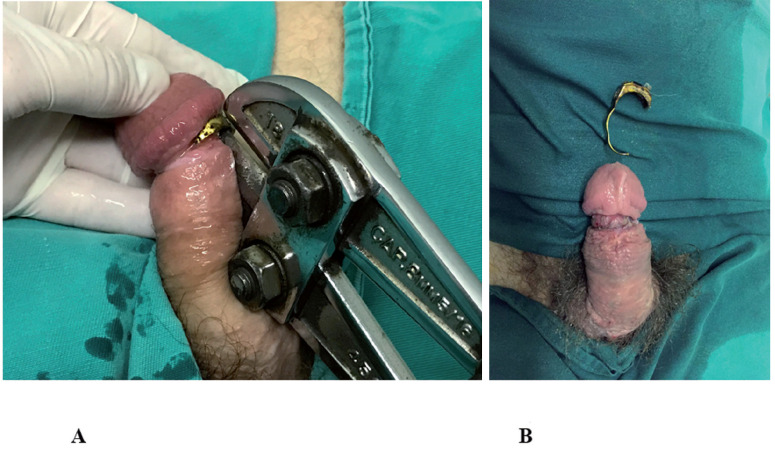
Patient with penile incarceration produced by metallic ring. Metallic ring removal through orthopedic cutting plier (A). Final appearance of the penile shaft after penile constrictor removal (B).

Unlike non-metallic penile constrictors that can usually be removed simply by incising the constriction device, metallic constrictor removal can be a challenge. In our series, the most commonly used device for removing metallic constrictor devices was the orthopedic cutting plier (55.5%), followed by the electric saw (33.3%) and dental drill (11.2%). There are several orthopedic pliers on the market whose cutting capacity can reach up to 0.9 cm thicknesses. The orthopedic cutting plier used in our institution was able to cut metal objects up to 0.8 cm thick (Figure-4). In three cases in which the metallic object had a thickness > 1.0 cm, the removal was done using an electric saw and dental drill manipulated by the fire brigade and the dentist on duty. Specialized teams that work with electrical devices are more proficient than urologists and should perform the extrication to avoid accidents (20). The use of a protective barrier between the foreign body and the penis is recommended, especially when electric devices are employed, to avoid iatrogenic injuries (21).

Acute urinary retention may occur due to urethral compression or injury produced by the foreign bodies. In our series, six patients (23%) presented with urinary retention that required bladder catheterization or supra-pubic catheter placement. A urethral evaluation may be necessary in cases of urethral fistula formation or suspected urethral stenosis. In this series, one (3.7%) patient developed a urethral fistula and two (7.4%) patients developed urethral stenosis. All patients were referred to the department of reconstructive surgery where they underwent urethroplasty through longitudinal penile skin flap. After the follow-up period, none of our patients reported lower urinary tract symptoms.

Depending on the severity of the injury caused by the constriction device, post-extraction complications can occur. Penile edema is the most common complication seen after constrictor extraction, with spontaneous resolution through reestablishment of venous and lymphatic drainage. In this series 25 (92.5%) patients developed penile edema with spontaneous resolution within 10 days. In exceptional cases, large penile edema can limit penile arterial blood flow, so color Doppler ultrasonography may aid in determining the vascular patency (21).

Lost or decreased penile sensation is an uncommon complication arising from the use of penile constrictor. It is probably related to the compression of the penile innervation exerted by the foreign body as well by the decrease of blood inflow in the affected area. In this series only 1 (3.7%) patient developed this condition whose resolution was spontaneous within 02 months, with no need of further treatments.

To our knowledge, this is the largest series published in the international literature.

## CONCLUSIONS

Penile strangulation is a rare urological emergency whose treatment must be immediate through the extraction of the foreign body, avoiding vascular impairments that can lead to serious complications. Most patients present with low-grade injuries and use penile constrictors due to erectile dysfunction. There are some treatment options, but depending on the case, their removal can be challenging. The use of specific tools for achieving penile release from constrictors is a fast, safe and effective method. Patients with urinary retention may require urinary diversion.
